# Frequency and spectrum of actionable pathogenic secondary findings in Taiwanese exomes

**DOI:** 10.1002/mgg3.1455

**Published:** 2020-08-14

**Authors:** Chieh‐Wen Kuo, Wuh‐Liang Hwu, Yin‐Hsiu Chien, Ching Hsu, Miao‐Zi Hung, I‐Lin Lin, Feipei Lai, Ni‐Chung Lee

**Affiliations:** ^1^ College of Medicine National Taiwan University Taipei Taiwan; ^2^ Department of Pediatrics National Taiwan University Hospital and National Taiwan University College of Medicine Taipei Taiwan; ^3^ Department of Medical Genetics National Taiwan University Hospital Taipei Taiwan; ^4^ Graduate Institute of Biomedical Electronics and Bioinformatics National Taiwan University Taipei Taiwan

**Keywords:** incidental finding, Taiwanese, whole‐exome sequencing

## Abstract

**Background:**

Exome sequencing has recently become more readily available, and more information about incidental findings has been disclosed. However, data from East Asia are scarce. We studied the application of exome sequencing to the identification of pathogenic/likely pathogenic variants in the ACMG 59 gene list and the frequency of these variants in the Taiwanese population.

**Methods:**

This study screened 161 Taiwanese exomes for variants from the ACMG 59 gene list. The identified variants were reviewed based on information from different databases and the available literature and classified according to the ACMG standard guidelines.

**Results:**

We identified seven pathogenic/likely pathogenic variants in eight individuals, with five participants with autosomal recessive variants in one allele and three participants with autosomal dominant variants. Approximately 1.86% (3/161) of the Taiwanese individuals had a reportable pathogenic/likely pathogenic variant as determined by whole‐exome sequencing (WES), which was comparable to the proportions published previously in other countries. We further investigated the high carrier rate of rare variants in the *ATP7B* gene, which might indicate a founder effect in our population.

**Conclusion:**

This study was the first to provide Taiwanese population data of incidental findings and emphasized a high carrier rate of candidate pathogenic/likely pathogenic variants in the *ATP7B* gene.

## Graphical abstract

This study was the first to study the frequency of reportable pathogenic/likely pathogenic variant in Taiwan, which was 1.86% in 161 individuals. In addition, a high carrier rate of candidate pathogenic/likely pathogenic variants in the ATP7B gene in Taiwan was also investigated in this study.

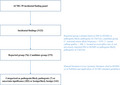

## 1 INTRODUCTION

Whole‐exome sequencing (WES) has become more feasible; therefore, more data are available to clinicians and may provide clues to previously obscure diseases. However, in addition to the genetic information being disclosed, another issue has developed, namely, the incidental discovery of secondary findings that are unrelated to the primary reason for ordering sequencing but that are of medical importance and could be used as a basis to improve a patient's health. In 2013, the American College of Medical Genetics (ACMG) recommended that a list of 56 genes should be reported by a laboratory to the ordering clinician, regardless of the indication for ordering sequencing, and that for most genes, only variants reported previously or predicted to be pathogenic should be reported (Green et al., 2013). The minimal 56‐gene list was revised to contain 59 genes in 2016 (Kalia et al., 2017). To identify pathogenic variants, which is also a challenging issue, the ACMG, the Association for Molecular Pathology (AMP) and the College of American Pathologists (CAP) jointly recommended the classification of variants into five categories based on set criteria: “pathogenic,” “likely pathogenic,” “uncertain significance,” “likely benign,” and “benign.” These criteria included typical types of evidence, such as population data, computational data, functional data, and segregation data (Richards et al., 2015). Recently, to improve the ACMG interpretation framework, the removal of criteria PP5 and BP6 (Biesecker & Harrison, 2018) and the development of a four‐step framework for criteria PS3 and BS3 (Brnich et al., 2020) were suggested.

Studies have proposed that the prevalence of pathogenic variants in actionable genes varies among different ethnic backgrounds. One study evaluated actionable pathogenic single‐nucleotide variants in 500 European‐ and 500 African‐descent participants, which showed frequencies of 3.4% and 1.2%, respectively (Dorschner et al., 2013). Another population‐based study including 196 Korean exomes revealed 11 pathogenic or likely pathogenic variants in 13 individuals (Jang, Lee, Kim, & Ki, 2015), while another Korean study involving 1303 exomes revealed 13 pathogenic and 13 likely pathogenic variants on the ACMG 59 gene list with a carrier rate of 2.46% (Kwak et al., 2017). A Japanese study of 2049 individuals undergoing whole‐genome sequencing reported 143 reported pathogenic variants for the 57 autosomal ACMG recommended genes and 21% of the individuals with at least one reported pathogenic allele according to public databases of pathogenic variations (Yamaguchi‐Kabata et al., 2018). Subsequently, data from 1005 whole exomes and genomes in Qatar disclosed a frequency of 0.59% actionable pathogenic or likely pathogenic variants in the population, which was lower than the frequencies previously reported in European and African populations (Jain, Gandhi, Koshy, & Scaria, 2018).

Although there is increasing attention being paid to reporting pathogenic variants in actionable genes, no study has been published to evaluate the prevalence rate of these variants in the Taiwanese population. Hence, this study analyzed WES data from 161 Taiwanese individuals and discussed the reporting of pathogenic/likely pathogenic variants on the ACMG 59 gene list in the Taiwanese population.

## 2 MATERIALS AND METHODS

### 2.1 Ethical compliance

This study was approved by the institutional review board of National Taiwan University Hospital (IRB NTUH 201703073RINB and 201505135RINA).

### 2.2 Patient enrollment

From 2017/6 to 2019/6, 166 unrelated patients suspected of having genetic disease who underwent exome sequencing were retrospectively analyzed. Of them, 80 underwent rapid trio‐exome sequencing (TruSeq Exome Kit), and 86 underwent single‐exome sequencing (Agilent V6) by a third‐party company; the results were analyzed by the Biomedical Genetic Laboratory of National Taiwan University Hospital. Five patients were excluded since the variants involved in the suspected diseases were included in the ACMG 59 gene list. Therefore, a total of 161 cases without parental data were further analyzed for incidental findings.

Three milliliters of whole blood was collected in an EDTA tube for DNA extraction using a Puregene DNA extraction system (Qiagen) after obtaining informed written consent. According to the informed consent, secondary findings were not disclosed to the participants.

### 2.3 Exome sequencing

Exome capture was performed using either the TruSeq Exome Capture Kit (Illumina) or the SureSelect Human All Exon V6 Kit (Agilent). Sequencing was performed using the NextSeq500 (Illumina) or HiSeq4000 (Agilent) kit. A 75‐bp paired‐end run was performed. A mean raw coverage over 100‐fold was obtained for each sample. Sequence alignment to the human reference genome (GRCh37) was performed using the Burrows–Wheeler aligner (BWA), and variant calling was performed using the Genome Analysis Tool Kit (GATK V3.5, Broad Institute) (Wang, Li, & Hakonarson, 2010).

### 2.4 Variant prioritization and interpretation

Variants were first annotated by wANNOVAR (http://wannovar.wglab.org/), followed by our in‐house pipeline (Richards et al., 2015; Wu et al., 2019). Variant pathogenicity from ClinVar (https://www.ncbi.nlm.nih.gov/clinvar/), the inheritance pattern from OMIM (https://omim.org/), and the allele frequency from the Taiwan biobank (https://taiwanview.twbiobank.org.tw/) were then added to the annotated file. The pathogenicity of variants was classified according to the American College of Medical Genetics and Genomics and the Association for Molecular Pathology guidelines (Biesecker & Harrison, 2018; Brnich et al., 2020; Retterer et al., 2016). Moreover, variants were reviewed based on information from different databases, such as the Human Gene Mutation Database (HGMD), ClinVar, Clinvitae, EmVClass, InterVar (Li & Wang, 2017), the M‐cap score (Jagadeesh et al., 2016), and VarSome (Kopanos et al., 2019), and for variants located in splicing regions, the Human Splicing Finder (HSF) (Desmet et al., 2009) and MaxEnt Scan (Yeo & Burge, 2004) were applied to evaluate the impact of alternating splicing sites.

### 2.5 Criteria for actionable gene list variants

**Figure 1:**
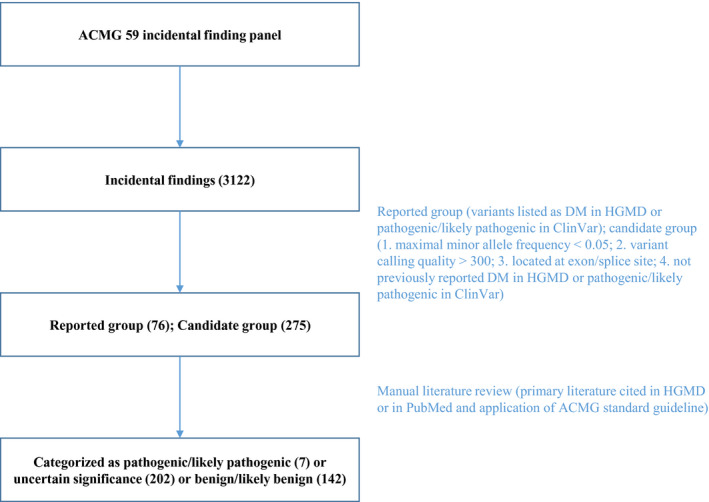
Algorithm of the selection of pathogenic or likely pathogenic variants. The exomes were first screened for variants from the ACMG 59 gene list. Then, the identified variants were included into the reported group or candidate group if they met the criteria. After application of all available lines of evidence and the ACMG standard guidelines, the identified variants were classified into three categories: pathogenic/likely pathogenic, uncertain significance, and benign/likely benign

As algorithm shown in Figure 1, the incidental findings listed as disease‐causing in the HGMD or pathogenic/likely pathogenic in ClinVar were included in the reported group. Additionally, the other variants were included in the candidate group if they met the following criteria: 1. a maximal minor allele frequency <0.05; 2. a variant calling quality >300; and 3. a location at an exon/splice site.

Afterward, the variants in both groups were reviewed according to all available lines of evidence, such as the primary literature cited in the HGMD and in PubMed. The ACMG standard guidelines (Richards et al., 2015) were applied to classify the variants into three categories: pathogenic/likely pathogenic, uncertain significance, and benign/likely benign.

## 3 RESULTS

### 3.1 Prevalence of variants from actionable gene list in the Taiwanese population

The data set analyzed in this research was composed of 161 whole exomes. As shown in Table 1 and Figure 1, a total of 3122 distinct variants of the 59 ACMG reportable genes were identified in the 161 exomes. Of these, 76 potentially pathogenic variants were classified as disease‐causing in the HGMD or pathogenic/likely pathogenic in ClinVar (reported group). In addition, 275 variants were identified based on the above criteria (candidate group).

**Table 1:** Classification of identified variants

Classification		Variants (N)
Total variants for 59 ACMG reportable genes		3122
Reported group		76
Candidate group		275
Pathogenic/likely pathogenic variants	Reported group	4
	Candidate group	3
	Sum	7
Uncertain significance variants	Reported group	37
	Candidate group	165
	Sum	202
Benign/likely benign variants	Reported group	35
	Candidate group	107
	Sum	142

In total, all 351 variants were reviewed according to all available lines of evidence, such as the primary literature cited in the HGMD or PubMed and the ACMG standard guidelines. The analysis revealed seven potentially pathogenic variants with four in the reported group and three in the candidate group, and *ATP7B* c.2804C>T was identified in two individuals. Detailed information on these seven potentially pathogenic variants is listed in Table 2.

**Table 2:** Pathogenic or likely pathogenic variants based on the ACMG guidelines

Variant information									Database							Frequency	
Gene	OMIM number	Chr: pos	Variant	AA change	dbSNP	ACMG interpretation result	Criteria	Disease‐associated	EmVClass	Clinvitae	M‐CAP score	ClinVar	HGMD	VarSome	InterVar	Maximum minor Allele frequency	Frequency in Taiwan
*MSH6*	120435	2:48026566	NM_000179.2:c.1444C>T	R482X	rs63750909	P	PVS1 PM1 PM2 PP3	Lynch syndrome	NA	P	PP	P	DM	P	P	0.0006	NA
*TSC2*	613254	16:2100489	NM_000548.5:c.225+2T>C		NA	LP	PVS1 PM2 PP3	Tuberous sclerosis syndrome	NA	NA	NA	LP	NA	P	NA	NA	NA
*SCN5A*	603830	3:38645439	NM_000335.5:c.1654G>C	G552R	rs3918389	LP	PS1 PM1 PM2 PP3	Brugada syndrome	NA	NA	PP	NA	NA	LP	LP	0.0002	NA
*ATP7B*	277900	13:52511739	NM_000053.4:c.3775_3776insAAAG	G1259Efs*14	NA	P	PVS1 PM1 PM2 PP3	Wilson disease	NA	NA	NA	NA	NA	P	NA	NA	NA
*ATP7B*	277900	13:52523835	NM_000053.4:c.2828G>A	G943D	rs779323689	LP	PS1 PM1 PM2 PP3	Wilson disease	NA	P/LP	PP	P/LP	DM	LP	LP	0.0019	0.00066
*ATP7B*	277900	13:52523859	NM_000053.4:c.2804C>T^a^	T935M	rs750019452	LP	PS1 PM1 PM2 PP3	Wilson disease	NA	P/LP	PP	P/LP	DM	LP	LP	0.0024	0.00198
*ATP7B*	277900	13:52532469	NM_000053.4:c.2333G>T	R778L	rs28942074	P	PS1 PS3 PM1 PM2 PP3	Wilson disease	P	P	PP	P	DM	P	LP	0.0023	0.00165

Abbreviations: DM, disease‐causing mutation; LB, likely benign; LP, likely pathogenic; NA, not available; P, pathogenic; PP, possibly pathogenic; VUS, variant of uncertain significance.

^a^ *ATP7B* c.2804C>T was identified in two individuals.

In total, eight participants, 4.97% of the participants analyzed, were identified as having pathogenic or likely pathogenic variants listed as incidental findings in the ACMG 59 gene list (Table 3). Of these participants, five (3.11% of the participants analyzed) had autosomal recessive variants in one allele, and three (1.86% of the participants analyzed) had autosomal dominant variants. Only pathogenic (known or expected) variants for dominant disease or biallelic variants for recessive disease met the ACMG criteria for reporting. Further investigation revealed that all the autosomal recessive variants were in the *ATP7B* gene, which highlighted the high carrier rate of Wilson disease in our population.

**Table 3:** Characteristics of participants with pathogenic/likely pathogenic variants

	Participant (n)	Percentage of participants (%)
Participants with pathogenic/likely pathogenic variants	8	4.97
Autosomal dominant	3	1.86
Autosomal recessive with two alleles found	0	0.00
Autosomal recessive with one allele found	5	3.11
X‐linked	0	0.00

### 3.2 Stop‐gain and frameshift variants

This study additionally evaluated stop‐gain and frameshift variants in the reported and candidate groups. A total of one stop‐gain and two frameshift variants were identified using the initial filtering criteria, which accounted for a lower percentage than missense variants. The stop‐gain variant in the reported group was *MSH6* c.1444C>T (p.R482X), which was predicted to be pathogenic. As shown in Table 2, six of seven databases reported this variant as pathogenic/likely pathogenic. The maximum minor allele frequency was 0.006. The *MSH6* c.1444C>T variant has been reported in several individuals with Lynch syndrome‐associated cancers (Baglietto et al., 2010; Hendriks et al., 2004; Okkels et al., 2012; Sjursen et al., 2010).

Of the two frameshift variants, *ATP7B* NM_000053.4:c.3775_3776insAAAG p.(G1259Efs*14) in one participant was predicted to be likely pathogenic (Table 2). In contrast, one frameshift variant, *MSH6* NM_000179.3:c.4068_4071dupGATT p.(K1358Dfs*2) (rs55740729), was identified in nine participants. The maximum minor allele frequency of this variant is 3.90%, which is not consistent with the disease presentation. The HGMD also reports this as a functional polymorphism. Therefore, by applying the PVS1, PP3, BS1, and BS2 criteria of the ACMG guidelines, this study classified this variant as a variant of uncertain significance, although several submitters classified it as benign/likely benign in ClinVar (Variation ID: 89518).

## 4 DISCUSSION

This study is the first to search for actionable pathogenic variants in the Taiwanese population. In this study, approximately 1.86% of the Taiwanese individuals had reportable pathogenic or likely pathogenic variants, that is, two AR alleles or one AD allele, as determined by WES. According to ACMG, pathogenic (known or expected) variants for dominant disease or biallelic variants for recessive disease should be reported.

In the published literature regarding incidental findings in WES and whole‐genome sequencing (WGS), the frequency of pathogenic/likely pathogenic variants has been variable, but approximately half are related to cardiovascular diseases (Amendola et al., 2015; Jain et al., 2018; Jang et al., 2015; Kwak et al., 2017; Olfson et al., 2015; Tang et al., 2018). By using the ACMG 59 gene list, a study involving 954 East Asian genomes found the frequency of pathogenic or likely pathogenic variants to be 2.5% (Tang et al., 2018). By using the ACMG 56 gene list, a study in Korea found a carrier frequency of actionable variants of 2.46% in 1303 individuals (Kwak et al., 2017). Another population‐based study including 196 Korean exomes identified 11 pathogenic or likely pathogenic variants in 13 individuals (Jang et al., 2015). In addition, a study involving 6503 individuals reported frequencies of actionable variants of 2% and 1.1% in European and African groups, respectively (Amendola et al., 2015). This study only enrolled 161 individuals, which is a smaller sample size than the above studies, which could have had an impact on the results. Additionally, there could be some rare polymorphisms that were identified as pathogenic/likely pathogenic variants in this study.

This study reported four of seven distinct pathogenic or likely pathogenic variants in the *ATP7B* gene, and the carrier rate was 3.11%. Previous studies showed variable prevalence rates across different ethnicities. One study in France estimated the prevalence rate of 1.5 cases per 100,000 (Poujois et al., 2018). A study in Taiwan found a prevalence rate of 1.81 cases per 100,000 (Tai et al., 2018); therefore, the heterozygous carrier rate was 0.85% based on Hardy–Weinberg equilibrium. In our study, we identified five out of 161 individuals who were carriers of an *ATP7B* pathogenic variant. Thus, the carrier frequency of WD‐related mutations was one in 32 (3.11%). This frequency is lower than that in France (3.2%) (Collet et al., 2018) but higher than those in Hong Kong (1.36%) (Mak et al., 2008), Korea (2%) (Park, Ki, Lee, & Kim, 2019), and the USA (1.1%) (Gao, Brackley, & Mann, 2019) according to molecular studies. This finding is consistent with Gao et al.’s observation that the East Asian population has the highest prevalence of Wilson disease, and the genetic prevalence of Wilson disease is greater than the epidemiological estimates (Gao et al., 2019). This finding is further supported by the fact that most of the pathogenic/likely pathogenic variants of *ATP7B* are found in the East Asian population in the Genome Aggregation Database (GnomAD), in which all eight individuals with variant c.2828G>A, 46 of 49 patients with variant c.2804C>T and all 37 patients with variant c.2333G>T were from the East Asian population. Considering those published studies and databases, the prevalence rate of Wilson disease and carrier rate of *ATP7B* gene mutations vary across different ethnicities. In particular, the prevalence rate and carrier rate are higher in East Asian countries, such as Taiwan, Korea, and China, and, surprisingly, in France. Therefore, given the history of the surrounding region, the higher carrier rate of mutated *ATP7B* gene variants could be due to the founder effect. Additionally, there could be some rare polymorphisms in the *ATP7B* gene. During the investigation, we found correctly classifying *ATP7B* variants to be challenging. Many rare variants still lack functional analysis, and some variants might be rare polymorphisms, especially in East Asia. Classifying those variants and deciding whether further reporting was warranted remain debatable issues among clinicians, and those decisions might influence future medical decisions for individuals. Thus, further studies on these rare variants in the *ATP7B* gene will be needed to answer these questions and guide laboratories with regard to the need to report those variants.

There were some limitations of this study. First, this study only included 161 Taiwanese participants, which may not fully represent the population. In the future, a multicenter study involving a larger number of participants will be necessary to evaluate the epidemiology of these pathogenic or likely pathogenic variants, especially those in the *ATP7B* gene.

In conclusion, we provided the first Taiwanese population data of incidental findings and highlighted a high carrier rate of candidate pathogenic/likely pathogenic variants in the *ATP7B* gene. This helps delineate the challenge of variant classification and benefits further interpretation.

## CONFLICT OF INTEREST

The authors declare no conflicts of interest.

## AUTHOR CONTRIBUTIONS

Wuh‐Liang Hwu, Yin‐Hsiu Chien, Feipei Lai, and Ni‐Chung Lee conceived the study. Ching Hsu and I‐Lin Lin performed the bioinformatics analysis. Chieh‐Wen Kuo and Miao‐Zi Hung carried out the variant analysis, interpretation, and literature reviews. Chieh‐Wen Kuo and Ni‐Chung Lee prepared, edited, and coordinated the manuscript. All authors approved the manuscript.

## References

[mgg31455-bib-0001] Amendola, L. M. , Dorschner, M. O. , Robertson, P. D. , Salama, J. S. , Hart, R. , Shirts, B. H. , … Jarvik, G. P. (2015). Actionable exomic incidental findings in 6503 participants: Challenges of variant classification. Genome Research, 25(3), 305–315. 10.1101/gr.183483.114 25637381 PMC4352885

[mgg31455-bib-0002] Baglietto, L. , Lindor, N. M. , Dowty, J. G. , White, D. M. , Wagner, A. , Gomez Garcia, E. B. , … Jenkins, M. A. (2010). Risks of Lynch syndrome cancers for MSH6 mutation carriers. Journal of the National Cancer Institute, 102(3), 193–201. 10.1093/jnci/djp473 20028993 PMC2815724

[mgg31455-bib-0003] Biesecker, L. G. , & Harrison, S. M. (2018). The ACMG/AMP reputable source criteria for the interpretation of sequence variants. Genetics in Medicine, 20(12), 1687–1688. 10.1038/gim.2018.42 29543229 PMC6709533

[mgg31455-bib-0004] Brnich, S. E. , Abou Tayoun, A. N. , Couch, F. J. , Cutting, G. R. , Greenblatt, M. S. , Heinen, C. D. , … Berg, J. S. (2020). Recommendations for application of the functional evidence PS3/BS3 criterion using the ACMG/AMP sequence variant interpretation framework. Genome Medicine, 12(1), 1–12. 10.1186/s13073-019-0690-2 PMC6938631 31892348

[mgg31455-bib-0005] Collet, C. , Laplanche, J.‐L. , Page, J. , Morel, H. , Woimant, F. , & Poujois, A. (2018). High genetic carrier frequency of Wilson’s disease in France: Discrepancies with clinical prevalence. BMC Medical Genetics, 19(1), 1–6. 30097039 10.1186/s12881-018-0660-3 PMC6086069

[mgg31455-bib-0006] Desmet, F.‐O. , Hamroun, D. , Lalande, M. , Collod‐Béroud, G. , Claustres, M. , & Béroud, C. (2009). Human Splicing Finder: An online bioinformatics tool to predict splicing signals. Nucleic Acids Research, 37(9), e67 10.1093/nar/gkp215 19339519 PMC2685110

[mgg31455-bib-0007] Dorschner, M. O. , Amendola, L. M. , Turner, E. H. , Robertson, P. D. , Shirts, B. H. , Gallego, C. J. , … Jarvik, G. P. (2013). Actionable, pathogenic incidental findings in 1,000 participants’ exomes. The American Journal of Human Genetics, 93(4), 631–640. 10.1016/j.ajhg.2013.08.006 24055113 PMC3791261

[mgg31455-bib-0008] Gao, J. , Brackley, S. , & Mann, J. P. (2019). The global prevalence of Wilson disease from next‐generation sequencing data. Genetics in Medicine, 21(5), 1155. 30254379 10.1038/s41436-018-0309-9

[mgg31455-bib-0009] Green, R. C. , Berg, J. S. , Grody, W. W. , Kalia, S. S. , Korf, B. R. , Martin, C. L. , … Biesecker, L. G. (2013). ACMG recommendations for reporting of incidental findings in clinical exome and genome sequencing. Genetics in Medicine, 15(7), 565. 23788249 10.1038/gim.2013.73 PMC3727274

[mgg31455-bib-0010] Hendriks, Y. M. C. , Wagner, A. , Morreau, H. , Menko, F. , Stormorken, A. , Quehenberger, F. , … Vasen, H. (2004). Cancer risk in hereditary nonpolyposis colorectal cancer due to MSH6 mutations: Impact on counseling and surveillance. Gastroenterology, 127(1), 17–25. 10.1053/j.gastro.2004.03.068 15236168

[mgg31455-bib-0011] Jagadeesh, K. A. , Wenger, A. M. , Berger, M. J. , Guturu, H. , Stenson, P. D. , Cooper, D. N. , … Bejerano, G. (2016). M‐CAP eliminates a majority of variants of uncertain significance in clinical exomes at high sensitivity. Nature Genetics, 48(12), 1581. 27776117 10.1038/ng.3703

[mgg31455-bib-0012] Jain, A. , Gandhi, S. , Koshy, R. , & Scaria, V. (2018). Incidental and clinically actionable genetic variants in 1005 whole exomes and genomes from Qatar. Molecular Genetics and Genomics, 293(4), 919–929. 10.1007/s00438-018-1431-8 29557500

[mgg31455-bib-0013] Jang, M.‐A. , Lee, S.‐H. , Kim, N. , & Ki, C.‐S. (2015). Frequency and spectrum of actionable pathogenic secondary findings in 196 Korean exomes. Genetics in Medicine, 17(12), 1007. 25856671 10.1038/gim.2015.26

[mgg31455-bib-0014] Kalia, S. S. , Adelman, K. , Bale, S. J. , Chung, W. K. , Eng, C. , Evans, J. P. , … Korf, B. R. (2017). Recommendations for reporting of secondary findings in clinical exome and genome sequencing, 2016 update (ACMG SF v2. 0): A policy statement of the American College of Medical Genetics and Genomics. Genetics in Medicine, 19(2), 249. 27854360 10.1038/gim.2016.190

[mgg31455-bib-0015] Kopanos, C. , Tsiolkas, V. , Kouris, A. , Chapple, C. E. , Aguilera, M. A. , Meyer, R. , & Massouras, A. (2019). VarSome: The human genomic variant search engine. Bioinformatics, 35(11), 1978. 30376034 10.1093/bioinformatics/bty897 PMC6546127

[mgg31455-bib-0016] Kwak, S. H. , Chae, J. , Choi, S. , Kim, M. J. , Choi, M. , Chae, J.‐H. , … Bang, Y.‐J. (2017). Findings of a 1303 Korean whole‐exome sequencing study. Experimental & Molecular Medicine, 49(7), e356. 28706299 10.1038/emm.2017.142 PMC5565953

[mgg31455-bib-0017] Li, Q. , & Wang, K. (2017). InterVar: Clinical interpretation of genetic variants by the 2015 ACMG‐AMP guidelines. The American Journal of Human Genetics, 100(2), 267–280. 10.1016/j.ajhg.2017.01.004 28132688 PMC5294755

[mgg31455-bib-0018] Mak, C. M. , Lam, C.‐W. , Tam, S. , Lai, C.‐L. , Chan, L.‐Y. , Fan, S.‐T. , … Chan, Y.‐W. (2008). Mutational analysis of 65 Wilson disease patients in Hong Kong Chinese: Identification of 17 novel mutations and its genetic heterogeneity. Journal of Human Genetics, 53(1), 55–63. 10.1007/s10038-007-0218-2 18034201

[mgg31455-bib-0019] Okkels, H. , Lindorff‐Larsen, K. , Thorlasius‐Ussing, O. , Vyberg, M. , Lindebjerg, J. , Sunde, L. , … Krarup, H. B. (2012). MSH6 mutations are frequent in hereditary nonpolyposis colorectal cancer families with normal pMSH6 expression as detected by immunohistochemistry. Applied Immunohistochemistry & Molecular Morphology, 20(5), 470–477. 10.1097/PAI.0b013e318249739b 22495361

[mgg31455-bib-0020] Olfson, E. , Cottrell, C. E. , Davidson, N. O. , Gurnett, C. A. , Heusel, J. W. , Stitziel, N. O. , … Bierut, L. J. (2015). Identification of medically actionable secondary findings in the 1000 genomes. PLoS One, 10(9), e0135193 10.1371/journal.pone.0135193 26332594 PMC4558085

[mgg31455-bib-0021] Park, H. D. , Ki, C. S. , Lee, S. Y. , & Kim, J. W. (2009). Carrier frequency of the R778L, A874V, and N1270S mutations in the ATP7B gene in a Korean population. Clinical Genetics, 75(4), 405–407. 19419418 10.1111/j.1399-0004.2008.01132.x

[mgg31455-bib-0022] Poujois, A. , Woimant, F. , Samson, S. , Chaine, P. , Girardot‐Tinant, N. , & Tuppin, P. (2018). Characteristics and prevalence of Wilson's disease: A 2013 observational population‐based study in France. Clinics and Research in Hepatology and Gastroenterology, 42(1), 57–63. 10.1016/j.clinre.2017.05.011 28648494

[mgg31455-bib-0023] Retterer, K. , Juusola, J. , Cho, M. T. , Vitazka, P. , Millan, F. , Gibellini, F. , … Bale, S. (2016). Clinical application of whole‐exome sequencing across clinical indications. Genetics in Medicine, 18(7), 696–704. 10.1038/gim.2015.148 26633542

[mgg31455-bib-0024] Richards, S. , Aziz, N. , Bale, S. , Bick, D. , Das, S. , Gastier‐Foster, J. , … Rehm, H. L. (2015). Standards and guidelines for the interpretation of sequence variants: A joint consensus recommendation of the American College of Medical Genetics and Genomics and the Association for Molecular Pathology. Genetics in Medicine, 17(5), 405. 25741868 10.1038/gim.2015.30 PMC4544753

[mgg31455-bib-0025] Sjursen, W. , Haukanes, B. I. , Grindedal, E. M. , Aarset, H. , Stormorken, A. , Engebretsen, L. F. , … Moller, P. (2010). Current clinical criteria for Lynch syndrome are not sensitive enough to identify MSH6 mutation carriers. Journal of Medical Genetics, 47(9), 579–585. 10.1136/jmg.2010.077677 20587412 PMC2976029

[mgg31455-bib-0026] Tai, C.‐S. , Wu, J.‐F. , Chen, H.‐L. , Hsu, H.‐Y. , Chang, M.‐H. , & Ni, Y.‐H. (2018). Modality of treatment and potential outcome of Wilson disease in Taiwan: A population‐based longitudinal study. Journal of the Formosan Medical Association, 117(5), 421–426. 28578978 10.1016/j.jfma.2017.05.008

[mgg31455-bib-0027] Tang, C.‐S.‐M. , Dattani, S. , So, M.‐T. , Cherny, S. S. , Tam, P. K. , Sham, P. C. , & Garcia‐Barcelo, M.‐M. (2018). Actionable secondary findings from whole‐genome sequencing of 954 East Asians. Human Genetics, 137(1), 31–37. 29128982 10.1007/s00439-017-1852-1

[mgg31455-bib-0028] Wang, K. , Li, M. , & Hakonarson, H. (2010). ANNOVAR: Functional annotation of genetic variants from high‐throughput sequencing data. Nucleic Acids Research, 38(16), e164. 20601685 10.1093/nar/gkq603 PMC2938201

[mgg31455-bib-0029] Wu, E.‐T. , Hwu, W.‐L. , Chien, Y.‐H. , Hsu, C. , Chen, T.‐F. , Chen, N.‐Q. , … Lee, N.‐C. (2019). Critical trio exome benefits in‐time decision‐making for pediatric patients with severe illnesses. Pediatric Critical Care Medicine, 20(11), 1021–1026. 31261230 10.1097/PCC.0000000000002068

[mgg31455-bib-0030] Yamaguchi‐Kabata, Y. , Yasuda, J. , Tanabe, O. , Suzuki, Y. , Kawame, H. , Fuse, N. , … Yamamoto, M. (2018). Evaluation of reported pathogenic variants and their frequencies in a Japanese population based on a whole‐genome reference panel of 2049 individuals. Journal of Human Genetics, 63(2), 213–230. 29192238 10.1038/s10038-017-0347-1

[mgg31455-bib-0031] Yeo, G. , & Burge, C. B. (2004). Maximum entropy modeling of short sequence motifs with applications to RNA splicing signals. Journal of Computational Biology, 11(2–3), 377–394. 15285897 10.1089/1066527041410418

